# Locally acquired malaria: a retrospective analysis of long-term surveillance data, European France, 1995 to 2022

**DOI:** 10.2807/1560-7917.ES.2024.29.41.2400133

**Published:** 2024-10-10

**Authors:** Hugues Delamare, Arnaud Tarantola, Marc Thellier, Clémentine Calba, Olivier Gaget, Paul-Henri Consigny, Frederic Simard, Sylvie Manguin, Elise Brottet, Marie-Claire Paty, Sandrine Houze, Henriette De Valk, Harold Noël

**Affiliations:** 1 Santé publique France, Direction des maladies infectieuses, Saint-Maurice, France; 2 Santé publique France – Île-de-France, Direction des régions, Saint-Maurice, France; 3 Sorbonne Université, AP-HP, Hôpital Pitié-Salpêtrière, Inserm, Institut Pierre Louis d’Epidémiologie et de Santé Publique, Paris, France; 4 Assistance Publique des Hôpitaux de Paris (AP-HP), Centre National de Référence du Paludisme, Paris, France; 5 Santé publique France – Provence-Alpes-Côte d'Azur, Direction des régions, Marseille, France; 6 Agence régionale de santé Auvergne-Rhône-Alpes, Lyon, France; 7 Institut Pasteur, Centre Médical, Paris, France; 8 MIVEGEC, University of Montpellier, IRD, CNRS, Montpellier, France; 9 HSM, University of Montpellier, IRD, CNRS, Montpellier, France; 10 Santé publique France – Auvergne-Rhône-Alpes, Direction des régions, Lyon, France; 11 Assistance Publique des Hôpitaux de Paris (AP-HP), Laboratoire de Mycologie et Parasitologie, Hôpital Bichat Claude Bernard, Paris, France

**Keywords:** Locally acquired malaria, *Plasmodium* spp., *Anopheles* spp., Epidemiology, Surveillance

## Abstract

**Background:**

In European France, the bulk of malaria cases are travel-related, and only locally acquired cases are notifiable to assess any risk of re-emergence.

**Aims:**

We aimed to contribute to assessing the health impact of locally acquired malaria and the potential of malaria re-emergence in European France by documenting modes of transmission of locally acquired malaria, the *Plasmodium* species involved and their incidence trends.

**Methods:**

We retrospectively analysed surveillance and case investigation data on locally acquired malaria from 1995 to 2022. We classified cases by most likely mode of transmission using a classification derived from the European Centre for Disease Prevention and Control. A descriptive analysis was conducted to identify spatial and temporal patterns of cases.

**Results:**

From 1995 to 2022, European France reported 117 locally acquired malaria cases, mostly due to *Plasmodium falciparum* (88%) and reported in Île-de-France (54%), Paris Region. Cases were classified as Odyssean malaria (n = 51), induced malaria (n = 36), cryptic malaria (n = 27) and introduced malaria (n = 3). Among the 117 patients, 102 (93%) were hospitalised, 24 (22%) had severe malaria and seven (7%) died.

**Conclusion:**

Locally acquired malaria remains infrequent in European France, with four reported cases per year since 1995. However, with the recent increasing trend in Odyssean malaria and climate change, the risk of re-emergence in non-endemic countries should be monitored, particularly in areas with autochthonous competent vectors. The vital risk of delayed diagnosis should make physicians consider locally acquired malaria in all patients with unexplained fever, especially when thrombocytopenia is present, even without travel history.

**Key public health message**

**What did you want to address in this study and why?**

In recent years, the number of locally acquired malaria cases attributed to transmission in healthcare institutions or airport transmission has increased in Europe. To inform control measures, we analysed two decades of notification data for locally acquired malaria in France to identify possible changes in the parasite's mode and frequency of transmission.

**What have we learnt from this study?**

Locally acquired malaria has remained constant in France between 1995 and 2022. However, since 2011, more cases have resulted from the bite of an infectious exotic mosquito imported in a plane, luggage or parcel (Odyssean malaria). For many cases, the source of infection cannot be identified, which illustrates the complexity of investigations. Among patients with locally acquired malaria, severe cases and death was more frequent than in imported cases.

**What are the implications of your findings for public health?**

Given the vital risk to the patient resulting from a delay in diagnosis, physicians should systematically consider the possibility of locally acquired malaria in patients presenting with unexplained fever, even without a travel history, especially when thrombocytopenia is documented. Disinsectisation of aircraft should be strictly enforced to prevent Odyssean malaria, and hygiene precautions is essential to contain cases of malaria in healthcare settings.

## Introduction

Malaria remains the most frequent vector-borne infectious disease worldwide with an estimated 249 million malaria cases and 608,000 deaths in 2022 [1]. Five species of *Plasmodium* parasites cause the disease in humans: *Plasmodium falciparum*, the most lethal type and the most prevalent on the African continent; *Plasmodium vivax,* mainly prevalent in countries outside of Africa; *Plasmodium ovale,* *Plasmodium malariae* and *Plasmodium knowlesi,* the latter occurring only in South-East Asia. Despite concerted efforts in malaria control, endemic malaria countries in Africa continue to bear a disproportionately high share of the global malaria burden, accounting for 94% of cases and 96% of deaths due to malaria globally [1].

Since the eradication of malaria in western Europe in the 1970s, cases are imported from endemic areas, but sporadic locally acquired cases are reported every year [2]. For epidemiological surveillance purposes, the European Centre for Diseases Prevention and Control (ECDC) classifies cases as indigenous if the transmission of the parasite occurred in the European Union (EU) and defines three main modes of transmission: airport or suitcase malaria (also called Odyssean malaria [3]), introduced malaria and induced malaria [4]. 

In the previous decade 2012 to 2022, < 0.2% of confirmed malaria cases in the EU were locally acquired [5]. Most locally acquired cases were reported in four countries: France, Greece, Italy and Spain [5-13]. In addition to sporadic cases, clusters of locally acquired *P. vivax* cases occurred repeatedly in Greece between 2009 and 2012 [14], while other countries reported sporadic cases involving mainly *P. falciparum* [6-8].

In European France, which excludes the overseas territories, malaria was eradicated in the 1960s with the last malaria focus reported in Corsica [15]. However, European France remains receptive to malaria, with *Anopheles* vectors mainly documented in Provence-Alpes-Côte d’Azur, Occitania [16] and Corsica [15]. Four local *Anopheles* mosquito species are considered malaria vectors with high potential [17]: *Anopheles hyrcanus* and *Anopheles plumbeus,* which have been detected in the southern natural park of Camargue, and members of the *Anopheles maculipennis* subgroup including *Anopheles atroparvus* and *Anopheles labranchiae* in Corsica. These species are mainly described as potential vectors for *P. vivax*. However, *An. plumbeus* is considered an efficient carrier of *P. falciparum* [18]. With 2,783 confirmed imported malaria cases in 2022, European France faces considerable vulnerability to malaria [5,19]. As a result, the risk of malaria re-emergence in European France cannot be ruled out.

Here we describe the characteristics of locally acquired malaria cases detected through mandatory notification in European France from 1995 to 2022. We focus on the modes of transmission and *Plasmodium* species involved to contribute to assessing the health impact of locally acquired malaria and the potential of malaria re-emergence in European France.

## Methods

### Surveillance system

The notification of locally acquired malaria cases, defined as the detection of *Plasmodium* parasites on a blood smear in a patient with no history of travel to a malaria-endemic area during the 12 months before the onset of symptoms, is mandatory in France. Malaria with long incubation period as observed with *P. malariae* and relapses due to *P. ovale* or *P. vivax* that occur more than 12 months after contamination do not meet the definition for locally acquired malaria. These malaria cases are either not reported as locally acquired by notifiers or ruled out by the investigations.

Physicians and microbiologists should promptly notify any case to the regional health agency authorities (ARS) and to Santé publique France, the French national public health agency (SpF), which undertake comprehensive investigations to identify the source and mode of infection. Medical laboratories are invited to systematically send the blood sample on which the diagnosis was made to the National Reference Centre for malaria (NRCm) for species confirmation and strain characterisation. The ARS, jointly with the SpF regional team, verifies whether the criteria for local acquisition are met and conduct the epidemiological investigations on possible modes of transmission and infection sources.

### Epidemiological investigations and mode of transmission

**Figure 1:**
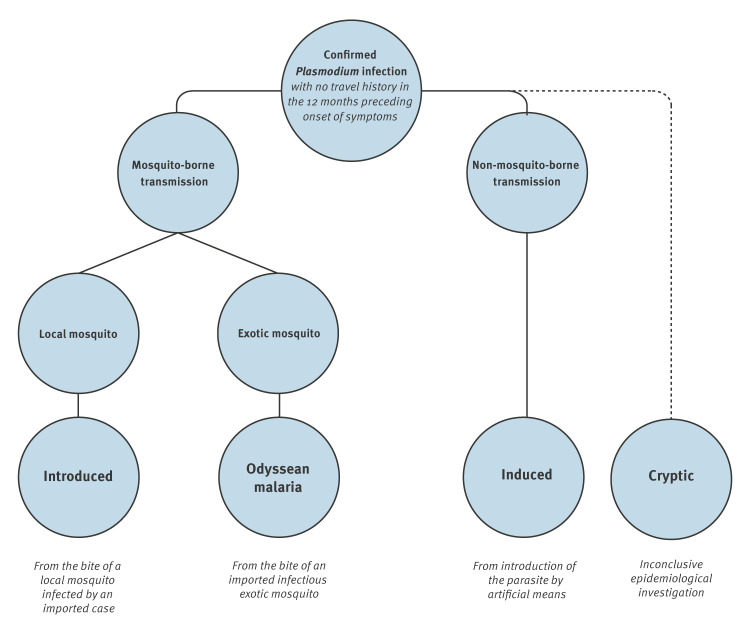
Classification of locally acquired malaria cases by mode of transmission

Based on the conclusion of the comprehensive investigations, we classified cases by most likely mode of transmission using a classification derived from the ECDC classification [4], displayed in Figure 1.

Introduction by artificial means refers to: accidental blood exposure, blood transfusion, mother-to-child transmission, organ transplant, or nosocomial transmission.

For epidemiological investigations, we first ruled out cases of malaria in whom the transmission of the parasite occurred in an endemic area. The investigations then assessed if transmission was attributable to artificial means: accidental blood exposure (‘needlestick malaria’ [20]), blood transfusion, mother-to-child transmission before or during delivery (perinatal), organ transplant, or whether it occurred during hospitalisation without specifying the treatment that led to contamination (nosocomial [21]), hence corresponding to a case of induced malaria [22].

If induced malaria was not evidenced, both Odyssean malaria, which results from the bite of an infectious exotic *Anopheles* mosquito imported by a plane, luggage or parcel from an endemic area [3], and introduced malaria, which results from the bite of a local mosquito infected by an imported human case, were investigated. In practice, if cases have not been inside or near an airport (usually within 5 km of the airport, but there was no official cut-off) or have not received a parcel from an endemic area, the investigation was focused on an introduced case. To focus on an introduced mode of contamination, it was essential to first confirm that the period was compatible with vector activity and that the vector was potentially present in the geographical area where the contamination occurred. If these criteria were met, a search for an imported index case was initiated and local authorities undertook entomological investigations. 

Finally, when the epidemiological investigation remained inconclusive and failed to identify an apparent mode of acquisition, cases were classified as cryptic malaria.

### Surveillance data

**Figure 2:**
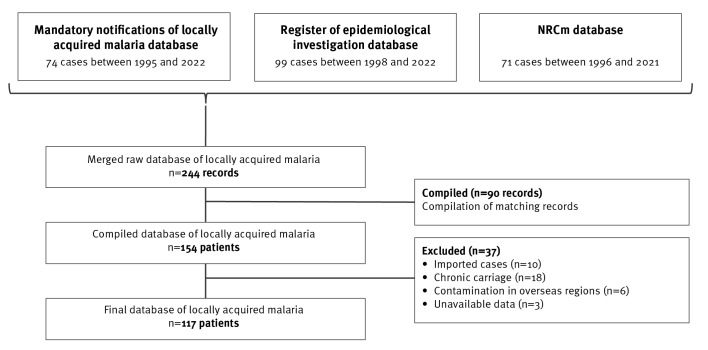
Flowchart of locally acquired malaria database

We retrieved locally acquired malaria cases from three different surveillance databases: the national database of mandatory notification of locally acquired malaria cases from 1995 to 2022, the register of epidemiological investigations of locally acquired malaria from 1998 to 2022, and the NRCm database from 1996 to 2021 (Figure 2).

NRCm: National Reference Centre for malaria.

All databases included information on: patient’s characteristics (age, sex, country of birth and *département* of notification), clinical presentation (date of onset, severity based on French guidelines [23], hospitalisation, outcome), diagnostics (e.g. *Plasmodium* species, *département* of the laboratory of diagnosis, date of diagnosis, sampling), suspected mode of transmission and conclusion of investigations. When the exact place of infection could not be identified, we used the *département* of notification as a proxy for place of infection.

We merged the three databases and compiled the information available in the databases for each case. When similar cases differed by minor date discrepancies or potential typing errors, we used collegial discussion based on records and correspondence reviews to conclude whether or not it was the same case. Cases infected in French overseas departments and territories or in other countries (imported cases) were excluded, along with cases with no information.

### Statistics

We conducted a descriptive analysis to identify spatial patterns and temporal trends of cases in European France. We used Fisher’s exact test for qualitative variables and a Kruskal–Wallis test and ANOVA for quantitative variables. The temporal trends were evaluated using a negative binomial generalised linear model. We did not conduct a linear regression due to the very small number of cases. The analysis was performed with RStudio Version 3.3.0+ (Posit PBC).

## Results

### Overall description

From 1995 to 2022, a total of 117 locally acquired malaria cases were reported in European France (Figure 2, Table), most involving *P. falciparum* (88%; n = 102). Cases were predominantly male (56%; n = 65) with a median age of 34.5 years (interquartile range (IQR): 23.8–45.3). Approximately half of the cases were born in an African endemic country (52%; n = 48), and 39 (42%) were born in France. Among the 117 patients, 102 (93%) were admitted to hospital. Clinically, 24 presented severe malaria, while 86 had an uncomplicated malaria. Overall, seven (7%) patients died (Table).

**Table:** Epidemiological characteristics of locally acquired malaria cases by mode of transmission, European France, 1995–2022 (n = 117)

Variables	Overall n = 117		Odyssean n = 51		Induced n = 36		Cryptic n = 27		Introduced n = 3		p value^a^
	n	% ^b^	n	% ^b^	n	% ^b^	n	% ^b^	n	% ^b^	
**Sex**											
Female	51	44	19	37	20	56	12	46	0	0	0.160^c^
Males	65	56	32	63	16	44	14	54	3	100	
Missing data	1		0		0		1		0		
**Median age in years (IQR)**	34.5 (23.8–45.3)		35.0 (27.5–41.5)		33.0 (0.0–58.0)		34.0 (26.0–44.5)		41.0 (35.0–50.0)		0.670^d^
Missing data	1		0		1		0		0		
**Place of diagnosis**											
Île-de-France	61	54	29	59	20	56	12	48	0	0	0.050^c^
Southern regions^e^	18	16	7	14	3	8	5	20	3	100	
Hauts-de-France	8	7	5	10	1	3	2	8	0	0	
Other regions	26	23	8	16	12	33	6	24	0	0	
Missing data	4		2		0		2		0		
**Place of birth**											
African malaria-endemic countries	48	52	21	58	9	29	18	78	0	0	< 0.001^c^
France	39	42	13	36	21	68	4	17	1	50	
European countries	4	4	2	6	1	3	1	4	0	0	
Other	1	1	0	0	0	0	0	0	1	50	
Missing data	25		15		5		4		1		
***Plasmodium* species**											
*P. falciparum*	102	88	49	96	25	71	26	96	2	67	0.002^c^
*P. malariae*	5	4	0	0	5	14	0	0	0	0	
*P. ovale*	5	4	1	2	4	11	0	0	0	0	
*P. vivax*	3	3	0	0	1	3	1	4	1	33	
*P. falciparum *and* P. malariae*	1	1	1	2	0	0	0	0	0	0	
Missing data	1		0		1		0		0		
**Severity**											
Severe malaria	24	22	9	18	9	27	6	24	0	0	0.670^c^
Uncomplicated malaria	86	78	40	82	24	73	19	76	3	100	
Missing data	7		2		3		2		0		
**Hospitalisation**											
No	8	7	3	6	3	9	2	8	0	0	0.900^c^
Yes	102	93	47	94	29	91	24	92	2	100	
Missing data	7		1		4		1		1		
**Outcome**											
Alive	90	93	46	98	23	82	19	95	2	100	0.080^c^
Deceased	7	7	1	2	5	18	1	5	0	0	
Missing data	20		4		8		7		1		
**Median time between symptom onset and diagnosis in days (IQR)**	4.0 (2.0–7.0)		4.5 (3.3–7.0)		2.0 (0.0–9.0)		3.0 (1.0–6.0)		6.0 (4.0–6.0)		0.590^f^
Missing data	19		5		12		2		0		

^a^ Statistical tests were used to compare the different modes of transmission. The ‘Overall' column was not included in the tests.

^b^ Due to rounding, the sum of certain percentages does not equal 100%.

^c^ Fischer exact test.

^d^ ANOVA test.

^e^ Southern regions include Provence-Alpes-Côte d’Azur, Occitania and Corsica.

^f^ Kruskal–Wallis test.

More than half of cases (54%; n = 61) were reported in Île-de-France, Paris Region. Cases were also reported in the Mediterranean regions of France (including Provence-Alpes-Côte d’Azur, Corsica and Occitania; 16%; n = 18), Haut-de-France (7%; n = 8) and in six other regions (23%; n = 26). Two regions, Bourgogne-Franche-Comté and Brittany, did not notify any locally acquired malaria cases. The median delay between onset of symptoms and biological diagnosis was 4 days, ranging from 0 to 37 days (data available for 98 cases).

### Mode of transmission

**Figure 3:**
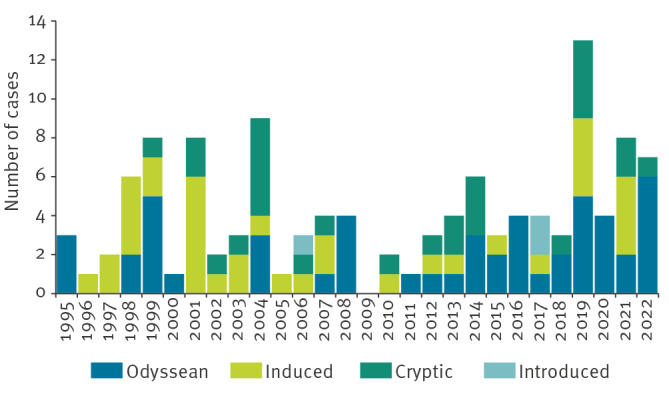
Locally acquired malaria cases by mode of transmission, European France,1995–2022 (n = 117)

Among the 117 cases reported between 1995 and 2022, 51 (44%) were categorised as Odyssean malaria, 36 (31%) as induced malaria, 27 (23%) as cryptic malaria and only three (3%) as introduced malaria (Table). There was no significant overall trend of locally acquired malaria incidence (proportion ratio: 1.04; 95% confidence interval (CI): 0.97–1.10; p = 0.30) (Figure 3).

Odyssean malaria is also referred to as airport, port or suitcase malaria.

**Figure 4:**
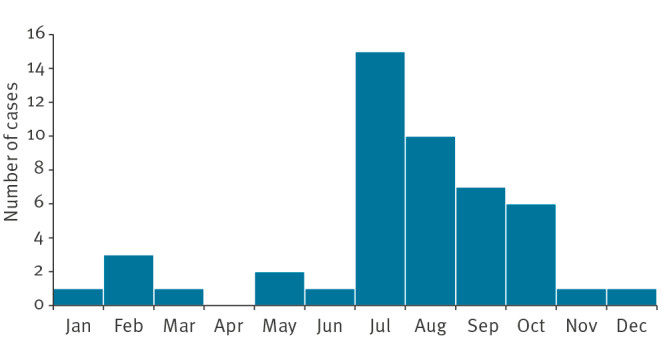
Odyssean malaria seasonality, European France,1995–2022 (n = 48^a^)

^a^ Three of the 51 Odyssean malaria cases had missing data on seasonality.

Among the 51 Odyssean malaria cases, 29 were attributed to a close geographical link with an airport, 16 to the handling of luggage or parcel and six to both. This close link with an airport was a recent visit, employment inside or usually within 5 km of the airport (5 km was usually used, but there was no official cut-off), or residence within a few km of the airport. Almost all cases were infected with *P. falciparum*, except for one infected with *P. malariae* and one with *P. ovale*. There was no report of Odyssean malaria involving *P. vivax.* More than half (n = 21) of the 36 patients for whom information were available were born in an African endemic country, and 13 cases were born in France. Two thirds were diagnosed in Île-de-France (n = 29). Sociodemographic characteristics of Odyssean cases did not differ significantly from other types of locally acquired malaria cases (Table). Nine cases developed severe malaria, all infected by *P. falciparum*, and one died. Incidence rose from an average of 1.2 cases a year with several case-free periods of 2–3 years between 1995 and 2010 to 2.7 cases reported yearly since 2011 (Figure 3). Odyssean malaria cases had a clear seasonality, with 32 cases diagnosed between July and September (Figure 4). There was a low increasing trend in the incidence of Odyssean malaria over the overall period (proportion ratio: 1.05; 95% CI: 1.00–1.09; p = 0.04).

### Induced cases

**Figure 5:**
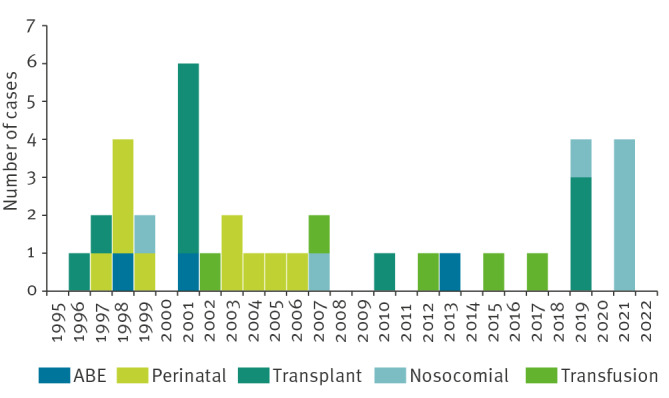
Evolution of induced cases, with detailed type of artificial contamination, European France 1995–2022 (n = 36)

Among the 36 cases of induced malaria, contamination occurred during transplant surgery for 11, during hospitalisation (nosocomial) for seven, during childbirth (perinatal) for 10, during blood transfusion for five, and following accidental blood exposure for three (Figure 5). No medical act reported in the patient files was found to potentially explain the contamination between patients in the seven nosocomial cases [21]. *Plasmodium falciparum* was less frequently involved (n = 25) in induced than in Odyssean malaria (n = 49). *Plasmodium malariae* and *P. ovale* were responsible for five and four infections, respectively (Table). There was one report of induced infection by *P. vivax*. Induced malaria cases were reported mainly in Île-de-France (n = 20) and were mainly born in France (n = 21). Nine patients, all infected with *P. falciparum*, developed severe malaria and five died. Among these five, three were infected during blood transfusion, one during transplant surgery and one during hospitalisation.

ABE: accidental blood exposure.

From 1995 to 2007, induced malaria was the principal mode of transmission of locally acquired malaria in European France with 23 cases reported. In the 10 years from 2008 to 2018, only five sporadic cases occurred, with several case-free years (Figure 3). In 2019 and in 2021, a total of eight cases were reported including five nosocomial infections (Figure 5). No perinatal malaria has been reported since 2007. There was no seasonality in the occurrence of induced cases. There was no significant trend of induced malaria incidence (proportion ratio: 0.98; 95% CI: 0.93–1.03; p = 0.40).

### Cryptic cases

Cryptic malaria cases (n = 27) with inconclusive epidemiological investigations were almost exclusively caused by *P. falciparum* (n = 26). For those with available information on place of birth, the proportion of patients born in Africa was significantly higher among cryptic malaria cases than other modes of contamination with 18 of 23 patients born in Africa vs nine of 31 and 21 of 36 among induced and Odyssean malaria cases, respectively (p < 0.001). No temporal trend was associated with the occurrence of cryptic malaria cases over the course of the study (proportion ratio: 1.04; 95% CI: 0.97–1.10; p = 0.30).

### Introduced cases

Introduced cases remained uncommon throughout the surveillance period with only three cases reported in the south of France. Two cases were potentially locally infected by *P. falciparum* while attending the same event in 2017 in Occitania. The other case was infected by *P. vivax* in 2006 in Corsica. All patients were hospitalised, experienced uncomplicated malaria, and all survived.

## Discussion

Locally acquired malaria has remained infrequent in European France, with a mean of approximately four cases annually since 1995. However, the challenges surrounding the diagnosis of malaria in patients without travel history leads to delayed treatment initiation and an increased risk of severity. Among the patients in our study, 22% had severe malaria and 7% died, far more than imported malaria patients during the same period, 6.8% and 0.4%, respectively [24]. Aside from the delay in diagnosing locally acquired malaria, induced malaria patients may more frequently have comorbidities and clinical features that could contribute to this elevated severity. To reduce severe malaria and prevent deaths, physicians need to be reminded about the possibility of locally acquired malaria in a patient with febrile thrombocytopenia, even without travel history [25].

In 2022, European France has the highest number of imported malaria cases among European countries with 2,783 cases, primarily from sub-Saharan Africa [24], followed by Germany with 768 cases [5]. However, among locally acquired malaria events linked to mosquito transmission, only three were attributed to local mosquitoes infected by imported cases, and no sustained transmission chains were observed. Since entomological investigations failed to detect any *P. falciparum*-infected *Anopheles* mosquitoes, and local *Anopheles* populations are not described as receptive to tropical strains of *P. falciparum* in European France, the conclusion of the two *P. falciparum* cases classified as introduced remains open to discussion.

Climate change with increasing temperatures, socioeconomic changes and population movements could modify the risk of autochthonous malaria re-emergence. Even if most *Anopheles* in European France seem unable to transmit tropical strains of *P. falciparum*, climate change could increase the vector competence of local *Anopheles* [26]. It could also turn certain areas into suitable habitats for imported *Anopheles* mosquitoes or for the establishment of invasive species such as *Anopheles stephensi* [27]. *Anopheles plumbeus* deserves special attention because of its reported role in the transmission of two introduced *P. falciparum* malaria cases in Germany [28]. This widespread *Anopheles* species may contribute considerably to increasing the malaria transmission risk of both *P. vivax* and *P. falciparum* in European France and elsewhere in central and western Europe [26,29]. As reported recently in the United States [30] or in earlier instances in Greece [14], clusters of introduced *P. vivax* malaria cases have occurred, and the conditions for future occurrence persist. This risk of occurrence is higher for *P. vivax* than for *P. falciparum* as local vectors have a history of transmitting *P. vivax.*

Odyssean malaria is the main mode of transmission of locally acquired malaria in European France. The share of Odyssean malaria among locally acquired cases in European France is higher than in other European countries where induced and introduced malaria prevails [6-8]. The substantial air traffic volume from malaria-endemic countries to European France could contribute to this difference [31]. A modelling study suggested that an outbreak cannot be sustained by transmission through imported vectors in Europe during the 21st century [32]. However, there has been a limited but significant increase in the yearly number of reported Odyssean malaria cases since 2010 in European France. Odyssean malaria has recently also been reported in Belgium [3] and in Germany [33,34]. This trend could be related to increased air traffic [35] and possibly to less stringent application of aircraft disinsectisation required by the International Health Regulations [36]. The higher temperatures measured during summer over the past decade, which are more suitable for the survival of exotic *Anopheles* mosquitoes, could also be a contributing factor to this increase [37]. Enforced vector surveillance and control measures at points of entry may decrease the risk of airport malaria [32] but would have little or no impact on mosquitoes imported in luggage or parcels from an endemic area.

Even if induced malaria cases are uncommon, artificial inoculation of the parasite can be avoided. Increased incidence of nosocomial cases in France in 2021 with no specific invasive procedure or other exposure identified could be linked to decreased attention, due to staff shortages or turnover, especially during the COVID-19 pandemic [21]. Prevention of healthcare-associated transmission requires that standard precautions be strictly implemented. Healthcare providers should be aware that hospital transmission of malaria is rare but possible [4].

The classification of cryptic malaria cases encounters complexity and limitations. In the absence of an exact mode of contamination, we cannot rule out that cryptic cases may include rare chronic and asymptomatic carriers [38,39]. Moreover, socio-administrative impediments, including linguistic challenges or deliberately omitted travel history were significant obstacles during the investigation of cryptic malaria cases. This may lead to inaccuracies, resulting in imported cases being misclassified as locally acquired cryptic cases [40].

Moreover, epidemiological and entomological investigations of locally acquired malaria cases are inherently complex due to incomplete data, limited size of events and potential delays. Concluding on the source and mode of transmission is challenging and such conclusions are based on multiple elements. It is sometimes impossible to conclude to a single mode of transmission. In such cases, investigators from various disciplines reach consensus on the most probable transmission mode. Finally, the completeness of our surveillance data could be affected by under-notification.

Due to reliance on historical surveillance databases, some variables were affected by missing values that could not be retrieved. Consequently, conclusions regarding the place of birth, the outcome and the median time between symptom onset and diagnosis may be heavily influenced by missing data. However, given that the outcome is a critical variable in the investigation, we believe that missing data are more likely to be associated with cases who remain alive rather than deceased cases. Even if the missing data corresponded to surviving patients, lethality of locally acquired malaria would still be considerably higher compared with imported malaria [24].

In addition, since locally acquired malaria is a rare disease in European France, the number of cases remained low throughout the study period. This resulted in an even smaller case count for each mode of transmission, potentially reducing the statistical power for comparisons. 

Recently, genomic analyses have proven to be an effective investigative tool for unravelling the mode of transmission and establish links between clustered cases. In cases of induced nosocomial malaria [21], these analyses can compare the *Plasmodium* parasite genotypes from an imported case and a nosocomial case who were hospitalised concurrently. In Odyssean malaria, genomics can provide insights into the phylogenetic and geographical origin of the parasite [34]. These new tools could lead to more accurate conclusions of investigations and should be used more routinely.

## Conclusion

Locally acquired malaria remains uncommon but should not be ruled out solely based on the absence of travel history. With the recent increasing trend in Odyssean malaria and climate change, the risk of malaria transmission in non-endemic countries should be closely monitored. The possibility of locally acquired malaria in patient without travel history should be highlighted to avoid misdiagnosis and delayed medical management, aiming to reduce severe malaria and prevent deaths.
